# Antimicrobial Potency and Bond Strength of Glass Ionomer Cement Following Cavity Disinfection with Er, Cr: YSGG Laser, and Iron Oxide Nanoparticles in Deciduous Molars

**DOI:** 10.12669/pjms.42.7.15717

**Published:** 2026-07

**Authors:** Zuhair Motlak Alkahtani

**Affiliations:** 1 Zuhair Motlak Alkahtani Department of Pediatric Dentistry and Orthodontic Sciences, College of Dentistry, King Khalid University, Abha, Saudi Arabia

**Keywords:** Cariously affected dentin, Glass ionomer cement, Iron oxide nanoparticles, Streptococcus mutans

## Abstract

**Objectives:**

To find out the effect of dentin surface disinfectants (Chlorhexidine (CHX), Er, Cr: YSGG laser (ECYL), and Iron oxide nanoparticles (Fe_3_O_4_NPs) on the inhibition of *Streptococcus mutans (S.mutans)* and the shear bond strength (SBS) of glass ionomer cement (GIC) to the caries-affected dentin (CAD) of primary molars.

**Methodology:**

The present in vitro trial was approved by King Khalid University. The study was completed in four months, August 15, 2025 to December 15, 2025. Forty mandibular primary second molar teeth exhibiting a score of four as per the International Caries Detection and Assessment System (ICDAS) were obtained. The criteria employed to procure CAD included visual inspection, evaluation of surface hardness, and staining. Subsequently, the teeth were distributed into four cohorts (n=10) based on different methods of disinfection. Group-1: No disinfection, Group-2: CHX, Group-3: ECYL, and Group-4: Fe_3_O_4_NPs. The agar diffusion test was employed to evaluate the antibacterial efficacy of the tested agents against S. mutans. GIC restoration was built up on the samples and artificially aged. The assessment of SBS and fracture patterns was conducted utilizing a universal testing apparatus and a stereomicroscope, respectively. The parametric One-Way ANOVA alongside Tukey’s post hoc tests were employed to enable comparisons among the tested groups, p < 0.05.

**Results:**

The widest zone of inhibition was presented by Group-2 (CHX) (15.95*±*1.54 mm). The narrowest zone was displayed by Group-1 (No disinfection) (6.24*±*1.13 mm). Group-3 ECYL (7.33*±*0.34 MPa) presented the highest bond SBS. Group-4 (Fe_3_O_4_NPs) exhibited the lowest bond strength (4.02±0.25 MPa).

**Conclusion:**

Er, Cr: YSGG laser demonstrated satisfactory antimicrobial potency and shear bond strength when used as a cavity sterilant on caries-affected dentin of primary molars.

## INRODUCTION

Dental caries in primary dentition remains a prevalent global health concern, with Streptococcus mutans identified as the principal causative microorganism. Preservation of primary teeth until their natural exfoliation is essential for maintaining proper masticatory function, guiding permanent tooth eruption, and supporting craniofacial development in pediatric patients.^1^ Glass ionomer cement (GIC) has emerged as a material of choice for restoring primary teeth, attributed to its unique properties including chemical adhesion to tooth structure, sustained fluoride release, coefficient of thermal expansion comparable to natural dentition, and inherent remineralization potential coupled with antimicrobial activity.^2^

Contemporary minimally invasive dentistry advocates selective removal of irreversibly infected dentin while preserving the remineralizable caries-affected dentin (CAD). However, this conservative approach presents restoration challenges, as CAD exhibits significantly compromised bond strength compared to sound dentin.^3^ This reduction stems from tubular occlusion by acid-resistant crystals and decreased substrate hardness. Additionally, residual cariogenic bacteria within CAD pose risks for restoration failure through secondary caries development, necessitating effective cavity disinfection protocols before restoration placement.^4^

Chlorhexidine (CHX), a cationic biguanide antiseptic with three decades of clinical application, demonstrates broad-spectrum antimicrobial efficacy through bacterial membrane disruption. Beyond its bactericidal properties, CHX inhibits matrix metalloproteinases (MMPs), potentially enhancing bond durability.^5^ Nasir et al. reported that CHX cavity disinfection did not improve composite bonding to primary molar CAD.^6^ However, its influence on GIC adhesion to CAD in primary dentition remains inadequately explored, warranting further investigation.^6^

Laser technology offers promising alternatives for dentinal disinfection. The erbium, chromium-doped yttrium-scandium-gallium-garnet (Er, Cr: YSGG) laser, operating at 2780 nm wavelength, demonstrates high absorption by hydroxyapatite and water, enabling effective bacterial elimination through hydrokinetic energy while simultaneously removing the smear layer.^7^–^9^ Its ablative mechanism creates microretentive surface patterns analogous to acid-etched morphology.^9^ Nevertheless, conflicting evidence regarding optimal laser parameters and their effects on bond integrity to primary tooth CAD necessitates additional research.

Recent nanotechnology developments have introduced innovative antimicrobial agents for restorative applications.^10^,^11^ Iron oxide nanoparticles (Fe_3_O_4_NPs), FDA-approved for medical imaging applications, exhibit potent antibacterial and antibiofilm properties. Preliminary studies suggest that Fe_3_O_4_NPs incorporation into dental adhesives maintains or enhances bond strength.^12^ However, their efficacy against *S. mutans* and impact on restoration bonding when employed as cavity disinfectants remain unexplored.

This investigation was designed to test the null hypothesis that no significant differences exist among various cavity disinfection protocols (CHX, Er, Cr: YSGG laser, and Fe_3_O_4_NPs) regarding antimicrobial effectiveness against *S. mutans* or shear bond strength of GIC to CAD compared to untreated controls. The study aimed to comprehensively evaluate these cavity disinfectants’ influence on bacterial suppression and adhesive performance when bonding GIC to caries-affected primary molar dentin.

## METHODOLOGY

The present in vitro trial was approved by King Khalid University. The study was completed in four months, August 15, 2025 to December 15, 2025. This laboratory investigation adhered to the Checklist for Reporting In-Vitro Studies (CRIS) guidelines to ensure methodological transparency and reproducibility.

### Ethical approval:

The present in vitro trial was approved by King Khalid University IRB-KKU-COD-ETH-2025-2026-81; dated August 1, 2025.

### Sample Selection and Preparation:

Forty extracted mandibular primary second molars were collected following extractions or physiological exfoliation.

### Inclusion criteria:

required teeth with cavitated carious lesions scoring four according to the International Caries Detection and Assessment System (ICDAS), indicating extensive dentinal involvement.

### Exclusion criteria:

encompassed teeth with structural defects (cracks, fractures), non-carious cervical lesions, hypoplastic defects, or previous restorative interventions. Following extraction, specimens were mechanically cleansed under running water to remove adherent soft tissues and debris, then stored in 0.5% chloramine-T solution at room temperature.^13^

### Caries-Affected Dentin Acquisition:

CAD was identified through multi-criteria assessment: visual-tactile examination using a sharp dental explorer to detect leathery consistency, and caries detector dye application (Kuraray Co., Ltd., Osaka, Japan). The dye was applied for 10 seconds, followed by water rinsing for 10 seconds. Light pink-stained regions indicating affected dentin were retained, while dark pink/red areas representing infected dentin were excavated using sterile round burs at low speed with intermittent water irrigation. The occlusal portions of teeth were sectioned perpendicular to the long axis using a water-cooled diamond disk mounted on a precision cutting machine. Exposed dentin surfaces were sequentially polished with 400-grit and 600-grit silicon carbide abrasive papers (Bosch, C355, Switzerland) under water lubrication to create a standardized smear layer. Prepared teeth were embedded in acrylic resin molds using auto-polymerizing acrylic (Probase Cold, Ivoclar Vivadent, Spain), leaving the dentin surfaces exposed and level with the mold surface.^13^

### Experimental Groups and Surface Treatments:

### Specimens were randomly allocated into four groups (n=10 per group):

#### Group-1 (Control):

CAD surfaces received no disinfection treatment, serving as baseline comparison.

#### Group-2 (Chlorhexidine):

2% chlorhexidine digluconate solution (Consepsis, Ultradent Products Inc., South Jordan, UT, USA) was actively applied using a microbrush for 20 seconds, followed by thorough water rinsing for 10 seconds and gentle air-drying for five seconds.

#### Group-3 (Er, Cr: YSGG Laser):

Erbium, chromium-doped yttrium-scandium-gallium-garnet laser (Waterlase iPlus, Biolase, California, USA) with MZ8 tip was positioned 2-mm from the substrate using a custom jig. Irradiation parameters included: wavelength 2780 nm, frequency 30 Hz, power 0.5 W, treatment duration 60 seconds, air pressure 65%, water pressure 55%. Following the laser application, surfaces were rinsed and air-dried.^13^

#### Group-4 (Iron Oxide Nanoparticles):

**Fig.1:**
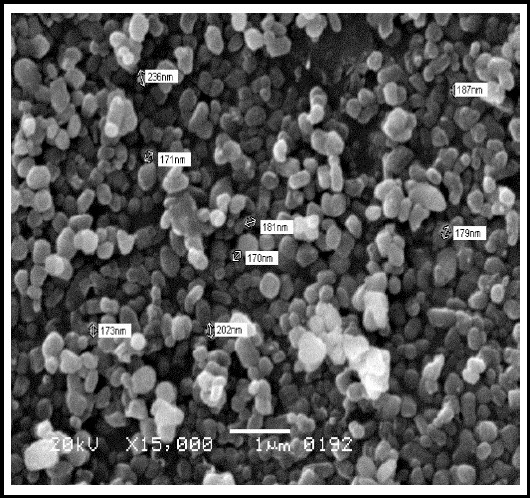
SEM image of Fe_3_O_4_NPs used for cavity disinfection of primary teeth.

Magnetite nanoparticles (Fe_3_O_4_NPs, particle size 25-50 nm (Fig.1), US Research Nanomaterials Inc., Houston, USA) were prepared as 1% aqueous suspension by dispersing 10 grams of powder in 1 liter of deionized water using ultrasonication for 30 minutes. Freshly prepared suspension was applied to CAD surfaces using a microbrush for 60 seconds, then rinsed and air-dried.^14^ Dynamic light scattering (DLS) characterization confirmed a bimodal size distribution, with a dominant nanoparticle population at a mean hydrodynamic diameter of 13.81 nm (90.8% relative intensity, SD: 6.53 nm) and a minor aggregate fraction at 259.8 nm (9.2% relative intensity); the PDI was 0.219, indicating acceptable monodispersity of the primary nanoparticle.

**Fig.2:**
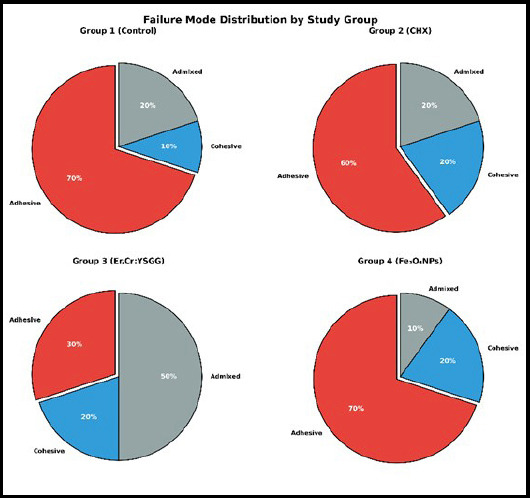
Failure mode distribution in study groups in percentages.

### Antimicrobial activity assessment:

Streptococcus mutans (ATCC 25175) was cultured on blood agar plates under anaerobic conditions (5-10% CO_2_) at 37°C for 48 hours. Antimicrobial efficacy was evaluated using the agar well diffusion method. Blood agar was autoclaved at 121°C (15 psi) for 20 minutes, then poured into sterile Petri dishes. After solidification, a standardized bacterial suspension (0.5 McFarland turbidity) was uniformly spread. Four wells (8 mm diameter) were created per plate using a sterile cork borer. Each disinfectant agent (50 μL) was dispensed into designated wells. Plates were incubated anaerobically at 37°C for 48 hours. Inhibition zones were measured in millimeters using digital calipers, representing the clear zone diameter around each well.^11^

### Glass Ionomer Cement Bonding Procedure:

CAD surfaces were conditioned with polyacrylic acid (Ketac Conditioner, 3M ESPE, Seefeld, Germany) for 10 seconds, rinsed thoroughly for 10 seconds, and blot-dried to maintain surface moisture. Ketac Molar Easymix (3M ESPE) was proportioned per manufacturer’s instructions. *One powder scoop:* One liquid drop), mixed for 30 seconds, and condensed into polyethylene Tygon tubes (internal diameter 5-mm, height 3- mm) positioned on prepared dentin. After five minutes, the tubes were removed, and specimens were stored at 37°C in 100% humidity for 24 hours.^2^

### Thermocycling protocol:

All bonded specimens underwent artificial aging through thermocycling (SD Mechatronik GMBH, Germany) for 10,000 cycles alternating between 5°C and 55°C water baths (dwell time 30 seconds, transfer time five seconds)

### Shear bond strength testing:

Specimens were mounted in a universal testing machine (Laryee Technology Co., Ltd., China) with a chisel-shaped loading rod positioned at the adhesive interface. Compressive shear load was applied at 0.5 mm/minute crosshead speed until failure. Bond strength was calculated: SBS (MPa) = Maximum load (N) ÷ Bonding area (mm²). Failure Mode Analysis. Fractured interfaces were examined under stereomicroscopy (AZ100M, Nikon, Japan) at 40× magnification. Failures were classified as: adhesive (interfacial separation), cohesive (failure within GIC or dentin), or mixed (combination patterns).

### Statistical analysis:

Data were analyzed using SPSS version 26.0. Normality was assessed using the Shapiro-Wilk test. One-way ANOVA with Tukey’s post-hoc test compared group differences. Statistical significance was set at p<0.05.

## RESULTS

### Inhibition zone:

Table-I displays mean±standard deviation values of the inhibition zone of *S. mutans* after applying different cavity disinfectants. The widest zone of inhibition was presented by Group-2 (CHX) (15.95±1.54 mm). Whereas the narrowest zone was displayed by Group-1 (No disinfection) (6.24±1.13 mm). Intergroup comparison analysis showed that Group-2, Group-3 (ECYL) (15.43±1.43 mm), and Group-4 (Fe_3_O_4_NPs) (15.77±1.33 mm) demonstrated no significant difference in inhibition zone thickness p>0.05

**Table-I:** The mean± standard deviation values of the inhibition zone of Streptococcus mutans after applying different cavity disinfectants.

Tested groups	Inhibition zone Mean ± SD (mm)	p-value!
Group-1: No disinfection (Control)	6.24±1.13 ^a^	<0.05
Group-2: CHX	15.95±1.54 ^b^	
Group-3: ECYL	15.43±1.43 ^b^	
Group-4: Fe_3_O_4_NPs	15.77±1.33 ^b^	

!ANOVA: Chlorhexidine (CHX), Er, Cr: YSGG laser (ECYL), Iron oxide nanoparticles (Fe_3_O_4_NPs).

The different superscript denotes a statistically significant difference (p<0.05), Post Hoc Tukey.

### SBS analysis:

Table-II demonstrates the SBS of conventional GIC to CAD after applying various cavity sterilants. Group-3 ECYL (7.33±0.34 MPa) presented the highest bond SBS. Whereas, Group-4 (Fe_3_O_4_) (4.02±0.25 MPa) exhibited the lowest bond strength. Intergroup comparative analysis discovered that Group-1 (No disinfection) (Control) (4.21±0.1 MPa), Group-2 (CHX) (4.55±0.23 MPa), and Group-4 demonstrated comparable bond strength outcomes. p>0.05.

**Table-II:** SBS of conventional GIC material to carious affected dentin after applying various cavity sterilants.

Tested groups	SBS Mean ± SD (MPa)	p-value!
Group-1: No disinfection (Control)	4.21±0.19 ^b^	<0.05
Group-2: CHX	4.55±0.23 ^b^	
Group-3: ECYL	7.33±0.34 ^a^	
Group-4: Fe_3_O_4_NPs	4.02±0.25 ^b^	

!ANOVA: Chlorhexidine (CHX), Er, Cr: YSGG laser (ECYL), Iron oxide nanoparticles (Fe_3_O_4_NPs).

The different superscript denotes a statistically significant difference (p<0.05), Post Hoc Tukey.

### Fracture pattern analysis:

Fig.1 presents the distribution of failure modes (in percentages) across the experimental groups. Admixed failures were observed most frequently in Group-3. While adhesive failures predominated in all other groups.

## DISCUSSION

This investigation tested the null hypotheses that various cavity disinfection protocols (CHX, Er, Cr: YSGG laser, and Fe_3_O_4_NPs) would demonstrate comparable antimicrobial efficacy and bond strength outcomes to untreated controls when applied to CAD in primary molars. The findings necessitated complete rejection of the first hypothesis, as all experimental disinfectants exhibited significantly superior antimicrobial activity against Streptococcus mutans compared to controls. The second hypothesis was partially rejected, since only Er, Cr: YSGG laser treatment significantly enhanced glass ionomer cement (GIC) bond strength relative to untreated substrates.

All tested disinfection protocols demonstrated substantial antibacterial efficacy against S. mutans. Iron oxide nanoparticles (Fe_3_O_4_NPs) exert bactericidal effects through multiple mechanisms, primarily reactive oxygen species (ROS) generation leading to lipid peroxidation, antioxidant enzyme depletion, and protein aggregation.^15^ Additionally, these nanoparticles induce intracellular iron overload, triggering oxidative stress culminating in bacterial cell death.^12^ These findings align with Ghorbanizadeh et al., who reported superior antibacterial performance of phosphate-functionalized Fe_3_O_4_ nanoparticles compared to chlorhexidine.^16^ The Er, Cr: YSGG laser achieves bacterial elimination through photothermal and photomechanical mechanisms. Laser energy absorption by intracellular water molecules generates localized hyperthermia exceeding bactericidal thresholds, denaturing essential bacterial proteins and enzymes.^17^,^18^ This mechanism’s effectiveness was corroborated by Türkün et al., demonstrating equivalent *S. mutans* reduction between laser irradiation and chlorhexidine application.^19^ Chlorhexidine’s antimicrobial action derives from its cationic structure, facilitating electrostatic binding to negatively charged bacterial cell walls, disrupting membrane integrity, causing cytoplasmic leakage, and inducing concentration-dependent bacteriostatic or bactericidal effects, consistent with observations by Deus et al.^20^ Bond strength analysis revealed Er, Cr: YSGG laser pretreatment significantly enhanced GIC adhesion to primary dentin compared to other protocols. This superior performance stems from laser-induced surface modifications creating microretentive patterns analogous to acid-etching, achieved through ablation and controlled substrate removal.^21^ The resulting increased surface area and roughness facilitate mechanical interlocking with restorative materials, though optimal outcomes require precise parameter calibration to prevent thermal damage or excessive ablation. These results corroborate findings by Nagarathna Chikkanarasaiah et al., who documented enhanced bonding following laser conditioning.^22^

Chlorhexidine application yielded bond strengths statistically comparable to controls, supporting previous observations by Yetkiner et al., who reported no interference with GIC adhesion.^23^ Glass ionomer cements bond through ionic exchange with hydroxyapatite, and 2% chlorhexidine does not disrupt these interactions in either sound or affected dentin.^23^ Multiple studies have confirmed chlorhexidine’s compatibility with GIC systems, contrasting with its documented interference with resin-dentin bonding, where the cationic agent creates a diffusion barrier preventing adequate monomer infiltration.^24^ However, literature remains limited regarding chlorhexidine’s specific effects on GIC bonding to CAD substrates in primary dentition.

Iron oxide nanoparticles, despite demonstrating potent antimicrobial properties, produced reduced bond strengths compared to controls. This unexpected outcome may result from iron-induced hydroxyapatite modifications. During nanoparticle application, iron ions potentially substitute calcium within the apatite crystal lattice, generating calcium-deficient apatite structures as documented by Sutter et al. These altered mineral phases may present unfavorable substrates for ionic bonding mechanisms essential to GIC adhesion, effectively creating a barrier that compromises adhesive performance.^25^ This hypothesis requires validation through detailed surface characterization studies examining mineral composition and bonding interface ultrastructure. Failure mode analysis substantiated quantitative bond strength data, revealing treatment-specific fracture patterns. Er, Cr: YSGG-treated specimens predominantly exhibited mixed failures, indicating enhanced interfacial adhesion where fracture propagation involved both adhesive and cohesive components.^17^ Conversely, control and alternative treatment groups showed predominantly adhesive failures at the restoration-dentin interface, confirming inferior bonding efficiency.

### Limitations

It includes the inability to replicate complex oral environmental factors: thermal cycling parameters, pH fluctuations, continuous salivary exposure, enzymatic activity, and masticatory loading patterns. The in vitro design, standardized specimen preparation, and limited sample size preclude direct clinical extrapolation. These findings require confirmation through additional laboratory investigations incorporating more sophisticated aging protocols and clinical trials before definitive recommendations. Nevertheless, results provide foundational evidence supporting Er, Cr: YSGG laser pretreatment as a promising protocol for enhancing GIC adhesion to caries-affected primary dentin while maintaining antibacterial efficacy.

### Novelty and contribution to medical literature:

This study provides the first comprehensive comparison of chemical (chlorhexidine), laser-based (Er, Cr: YSGG), and nanotechnology-based (iron oxide nanoparticles) cavity disinfection methods specifically in primary molar CAD. Unlike previous research focusing on permanent teeth or single outcomes, this investigation integrates antimicrobial efficacy and glass ionomer cement bond strength assessment—both critical for pediatric restoration success.

## CONLCUSION

Er, Cr: YSGG laser displayed satisfactory antimicrobial potency and shear bond strength outcomes when applied as a cavity sterilant on carious affected dentin of primary molars. However, more studies are required to confirm these outcomes.

### Recommendations:

This study pioneers iron oxide nanoparticle application in primary dentition, revealing important limitations that guide future formulation development. By addressing the most used pediatric restorative material (GIC) with standardized caries assessment, this research fills a critical knowledge gap, providing evidence-based protocols for minimally invasive pediatric dentistry and supporting tissue-conservative approaches in children.
